# Efficiency of Our Medical Schools

**Published:** 1872-12

**Authors:** 


### EFFICIENCY OF OUR MEDICAL SCHOOLS.

During the last few years the question of Medical Education in its relation to our professional wants has been attracting a large share of attention. There has been a prevailing impression that our systems do not answer the requirements of the times in which we live. Little has been done, however, either towards defining faults or remedies. Recently the subject has gained new interest from important changes that have taken place in one of our sister colleges, and careful inquiry is now being made into the practical working and comparative efficiency of medical systems here and elsewhere. While much may be learned by instituting proper comparisons, we do not propose to attempt them at present, nor shall we advocate such changes as would materially alter the present status of our schools. Our aim is to expose what seems to us to be prominent defects, and to submit what we conceive to be appropriate remedies, offering such amendments to our present schemes of instruction as may serve to supplement rather than supercede them. We do not wish to hold up any one pattern for approval, but simply to call attention to certain principles which seem to be at the basis of sound medical education. As an instance, the profession will certainly agree with us that our colleges should furnish us with thorough theoretical and practical instruction in all branches of medicine practised among the profession at large; and yet we know that even in our largest cities, where cultivation and refinement rank highest, and where the demands on our profession are as exacting as in any part of the civilized world, such is not the case. We know that our colleges often fail to provide practical instruction for their pupils in departments where practising physicians have developed for themselves specialities that are the means of vastly increasing our field of usefulness, and enable us sometimes to save life. We might enumerate examples showing this, but will merely specify a single instance.

It is notorious that the discovery of the Laryngoscope has been made of valuable service to us, and we may now contend successfully with diseases that were once looked upon as intractable. Now while this is true, it is equally true that Laryngoscopy is not generally recognized, and in fact is not taught to-day in a single medical college in this city.

Again, the sphere of instruction might be extended further in the direction of other allied sciences. A well organized school of medicine should not only include in its curriculum the practical branches which every practitioner needs in his private practice, but also the accessory ones, that bring him into relation with the public at large.

We ought to have sound practical instruction in Medical Jurisprudence for our graduates. For our students we should have courses on Embryology as well as on Histology and Pathology. The principles of Hygiene, too, with Sanitary laws, should have appropriate places in our systems.

Another point of importance is, that the larger departments in medicine should be subdivided in such a way that the entire ground may be fairly and equally covered by the instructors.

In urging the necessity of this last point, we are fully aware of the objection that the student's time is already fully occupied with the courses recommended for him to pursue. We would merely reply, that whatever innovations have been suggested might be made entirely consistent with the operation of the present system; for while every student might have the facilities offered him of getting a more thorough medical education, no new difficulties need be interposed between him and his diploma.

We do not wish, however, to defend a system that entitles the graduate, by virtue of his diploma, to practice whatever branch of medicine he may choose, when in very many of them he is ignorant of their first principles. He can hardly have any practical acquaintance with Ophthalmology or Otology, or a half-dozen other special branches of medicine. In fact, whatever he learns of these studies is not derived from the regular course of lectures at all, but from independent clinical or didatic instruction, and he is rarely questioned on them by his medical examiners.

The recent graduate, recognizing this, is compelled to begin, as it were, a new course of study before he can fit himself for taking rank among the really well educated men of his profession, or before he can be safely trusted with the lives of the community.

Our method of instruction, too, is faulty. Take, for example, many of our really excellent lectures on anatomy. The thoroughness in description and almost absolute accuracy in each detail leave hardly anything to be desired as far as truthfulness in the representation is concerned. The practitioner or the ripe anatomist may be delighted; but is it not wholly unpalatable for the beginner? And yet students who have never seen a human bone before, and others who have been studying anatomy two full years, have but this single course of lectures offered them. What may be said of Anatomy may be said of Chemistry. How much more rational would it be to supply beginners with rudimentary instruction—the aim being first to insure a proper grounding, as, for instance, by dissecting-room work done under capable demonstrators, and courses in the chemical laboratory under assistants. Practical experience gained in this way might be supplemented by short didatic courses, presenting such facts in connection with the work already done, as might be readily understood and digested. So, too, Internal medicine might be taught clinically, until the student has a sufficient practical familiarity with disease, and then theoretical instruction would be doubly useful.

In a similar way the second and third year student might be furnished with material suited to the particular characters of their wants. In those departments where two full terms might well be devoted to the subject, a feasible plan would be for one instructor to conduct the longer and more complete course, while an assistant takes the shorter or more elementary one.

If the same plan were carried out through the entire course, the relative importance of each branch being impressed upon the student, and the system so contrived that he could advance by easy and natural stages from the subordinate to the higher studies, is it not probable that better practitioners would be made ?

Such a system as is here suggested would require a larger corps of instructors, but it would be more efficient. Of course some reoganzation would have to be made of our medical bodies. A plan similar to that instituted at some German Universities might be adopted. In the Anatomical Department, for example, besides the regular Professor, there might be a co-ordinate Professor, lecturing during the same course, and on the same subject, but to a less advanced class. In some very comprehensive department, there might be added an extraordinary Professor to the regular and co-ordinate. It would be the privilege of such an extraordinary Professor to supplement the regular course of lectures by supplying certain deficiencies in it.

Thus Military Surgery and Surgical Pathology might be advantageously attached to the chair of General Surgery; in addition, there might be a corps of Private Instructors duly commissioned to instruct in certain branches, and prepared to assume the position of extraordinary professors, should any vacancy occur or new chair be instituted.

Finally, to each chair a sufficient number of assistants might be attached, and the numbers made reasonably large, and from them the Private Instructors might be recruited. The advantages of such an organization are evident.

The college will retain about itself a large class of young men who will be stimulated to exertion by a generous rivalry. From these it will be able to select for advancement to higher positions those who have shown proofs of their ability. Many who now go abroad, to pursue special studies, will be retained at home, and we shall not be mortified by knowing that our medical men are obliged to flock to foreign universities to get an adequate education.

It may be said the system is cumbrous. But it can be shown to have worked well, and to have answered the requirements. Those who have attended the courses in the Allgemeine Krankenhaus in Vienna readily see how possible it is to furnish instruction in every branch of medicine. Multitudes of short courses are made up from time to time, and the practitioner or student can get a rapid and condensed, or long and thorough course on any subject whatever.

There are many good reasons why we do not imitate any such plan. In many of our dispensaries and public institutions, outside of our colleges, however, we might furnish good clinical instruction; and specially commissioned attachés of our colleges might be empowered to conduct regular and systematic courses.

The objection that, pecuniarily, the plan would fail, has been urged as a strong argument against it. But why should we not have as much protection in medicine as in other sciences. Has not the State of Massachusetts liberally sustained a certain Institute at one of its principal universities, and encouraged the study of the Natural Sciences, when the laborers in those fields were few; and has not private liberality often sustained chairs in our Medical Schools? It is not likely that, ten years hence, the cultivation of the advanced studies of medicine will be any more self-supporting than they are now. They never have been anything but a burden on the State, or on individuals, and yet to them we owe many of the discoveries that have thrown light on our diagnosis and treatment.

If the progress of medicine is to be sustained, it must be in a great measure through support from without. We hope this idea will receive proper encouragement, and that assistance from any quarter will be sought for and thankfully received.

We have thrown out these suggestions with the view of attracting the attention of those concerned with the future education of our medical men; their practical acquaintance with teaching will enable them to judge how far such changes are feasible in the present state of medical science in America. *Medical Record.*

